# Functional and Compositional Stability of Bacterial Metacommunities in Response to Salinity Changes

**DOI:** 10.3389/fmicb.2017.00948

**Published:** 2017-06-08

**Authors:** Mercè Berga, Yinghua Zha, Anna J. Székely, Silke Langenheder

**Affiliations:** ^1^Department of Ecology and Genetics/Limnology, Evolutionary Biology Centre, Uppsala UniversityUppsala, Sweden; ^2^Biological Oceanography, Leibniz Institute for Baltic Sea Research WarnemündeRostock, Germany

**Keywords:** adaptation, bacteria, community assembly, community composition, community function, disturbances, resistance, salinity

## Abstract

Disturbances and environmental change are important factors determining the diversity, composition, and functioning of communities. However, knowledge about how natural bacterial communities are affected by such perturbations is still sparse. We performed a whole ecosystem manipulation experiment with freshwater rock pools where we applied salinity disturbances of different intensities. The aim was to test how the compositional and functional resistance and resilience of bacterial communities, alpha- and beta-diversity and the relative importance of stochastic and deterministic community assembly processes changed along a disturbance intensity gradient. We found that bacterial communities were functionally resistant to all salinity levels (3, 6, and 12 psu) and compositionally resistant to a salinity increase to 3 psu and resilient to increases of 6 and 12 psu. Increasing salinities had no effect on local richness and evenness, beta-diversity and the proportion of deterministically vs. stochastically assembled communities. Our results show a high functional and compositional stability of bacterial communities to salinity changes of different intensities both at local and regional scales, which possibly reflects long-term adaptation to environmental conditions in the study system.

## Introduction

Natural communities are open systems that are influenced by local processes (e.g., competition, niche differentiation, or predation), by regional processes (e.g., dispersal from/to a locality), as well as by historical factors (e.g., speciation or colonization events) (Cornell, 1999; Hillebrand and Blenckner, 2002). These processes determine the diversity and composition of communities and potentially their response to environmental change and disturbances. Communities can respond differently to disturbances depending on the type, intensity and frequency of the disturbance as well as on the capacity of the different species to tolerate them (Sousa, 1984). This relates to central concepts of stability including (i) resistance, which is the ability of a community to withstand a disturbance, (ii) recovery, which is the ability of a response variable to return to the initial state, and (iii) resilience (more specifically engineering resilience), which is the rate of recovery of a community to pre-disturbance conditions (Pimm, 1984). During the last decade, an increasing number of studies have also looked at effects of disturbances on bacterial community composition (BCC) and functioning in diverse set-ups, ranging from experiments at the lab-scale to field studies, investigating changes along environmental gradients. The consensus of this work that has been summarized by Allison and Martiny (2008) and Griffiths and Philippot (2013) for soils and more generally by Shade et al. (2012) is that bacterial communities are in most cases not resistant to disturbances. Moreover, compositional changes concur with functional changes in some cases, but not in others, indicating that compositional and functional resistance and resilience are not necessarily linked. For example, functional redundancy among bacterial taxa can result in a situation where composition changes in response to the disturbance whereas functioning remains stable. Whether or not functional redundancy is found will also be influenced by the type of function that is measured (Langenheder et al., 2006; Peter et al., 2011) and the strength and type of the disturbance that is applied (Langenheder et al., 2005; Berga et al., 2012; Comte et al., 2013). Finally, disturbances may also reduce species richness (alpha-diversity) of bacterial communities, which might have negative consequences for the magnitude and stability of ecosystem processes. There is, however, contrasting evidence if this is actually the case (Peter et al., 2011; Delgado-Baquerizo et al., 2016; Roger et al., 2016).

Generally spoken, disturbance strength (intensity) is a key feature that will determine how communities of macro- as well as microorganisms respond to the disturbance (Sousa, 1984; McCabe and Gotelli, 2000; Berga et al., 2012; Shade et al., 2012; Gibbons et al., 2016). Disturbance strength can influence the community response by altering mechanisms such as physiological plasticity, adaptation, species extinctions, or changing community composition (Sousa, 1984; Lake, 2003; Griffiths and Philippot, 2013). To date, there are only few empirical studies that have investigated how disturbance strength influences compositional turnover or alpha-diversity (species richness) in bacterial communities. Berga et al. (2012) found gradual changes in bacterioplankton composition in response to a salinity disturbance of increasing intensity. Gradual reductions in richness in response to increasing disturbance frequencies in soil bacterial communities have also been observed (Kim et al., 2013). In contrast, Gibbons et al. (2016) found evidence for unimodal disturbance–diversity relationships in response to disturbance intensity and frequency of two disturbance types (biomass removal, UV exposure) in a complex culture system. Their results supported the Intermediate Disturbance Hypothesis, which predicts that diversity in communities is highest at intermediate rates of disturbance frequency and intensity (Connell, 1978).

Disturbances can also affect communities at the regional scale and change beta- and gamma-diversity as well as community assembly mechanisms, in particular whether communities are stochastically or deterministically assembled. If communities are stochastically assembled their composition is determined by random birth, death as well as immigration and emigration events, whereas species sorting by local environmental conditions leads to deterministically assembled communities (Nemergut et al., 2013). In general, effects of disturbances at the regional scale are less studied and focused mainly on laboratory experiments with simple microbial model communities (e.g., Cadotte, 2007) or field studies/experiment with larger organisms (Chase, 2007; Lepori and Malmqvist, 2009; Vanschoenwinkel et al., 2010). The consensus of these studies is that even at this scale disturbance strength appears to be important and it has been shown that beta- and gamma-diversity peak at intermediate disturbance strength (Cadotte, 2007), where co-existence of species was highest. Experiments performed under more realistic conditions in the field showed that strong disturbances affecting all local communities in a region or metacommunity decrease beta-diversity (Chase, 2007; Lepori and Malmqvist, 2009; Vanschoenwinkel et al., 2010) and this effect has also been found for bacterial communities in soils as a response to a disruption related to land conversion (Rodrigues et al., 2013). Such decreases in beta-diversity can occur as a result of strong deterministic effects of the disturbance, because it filters out species that are unable to resist harsh environmental conditions (Chase, 2007; Jiang and Patel, 2008). However, several studies with microbial communities have also shown that the timing of the sampling in relation to the disturbance is important and that the importance of stochastic assembly processes peaks at early stages after the disturbances, whereas deterministic processes became more important at later stages of the succession processes (Ferrenberg et al., 2013; Zhou et al., 2014; Zhang and Zhang, 2015).

The response of bacterial communities to disturbances may also be influenced by dispersal (e.g., Shade et al., 2012; Vuono et al., 2016). For example, dispersal can introduce new species to local communities that are specialized to the disturbance conditions or enable the re-colonization by taxa that became locally extinct in response to a disturbance, thereby enhancing community resilience. Most studies that have tested the resistance and resilience of complex bacterial communities have been done at the scale of laboratory experiments (Shade et al., 2012). They do often have the disadvantage that they preclude natural dispersal sources as well as recruitment processes from internal seed-banks, both of them being important features of natural communities (Lennon and Jones, 2011). In general, field experiments addressing how bacterial communities respond to disturbances are limited and have mostly focused on local communities (see Jones et al., 2008; Shade et al., 2012). Shade et al. (2011) performed an experiment with planktonic bacterial communities in lakes where they studied the effect of lake mixing and found that bacterial communities were resilient to nutrient and oxygen changes caused by the mixing. Jones et al. (2008) studied the response of freshwater bacterial communities to typhoon-induced water mixing and concluded that bacterial communities were able to recover fast. However, these studies were not able to address the effects of disturbance strength on bacterial communities in multiple ecosystems, i.e., across space, at the same time. We also lack understanding about how both compositional and functional stability are affected by differences in disturbance intensity at the field scale. For this purpose, rock pools are ideal natural model systems since they are highly variable systems where disturbances occur naturally and frequently (Srivastava et al., 2004; Jocqué et al., 2007; Langenheder et al., 2012). Evaporation and reduced precipitation cause drying events as well as increases of salinity by concentrating the salt that pools receive by sea-spray. Salinity causes differences in BCC among rock pools (Langenheder and Ragnarsson, 2007; Langenheder et al., 2012) and is generally a very strong environmental (Lozupone and Knight, 2008) and evolutionary filtering force (Logares et al., 2009). Moreover, the fact that disturbances occur frequently and at different intensities offers the opportunity to study whether adaptation to these conditions occurs. Finally, rock pools are small, which makes it easy to manipulate them, and they are located close to each other so that they are all influenced by the same climate regime. They are filled during rainfall and thus influenced by bacteria dispersed with rain.

Here we performed a whole ecosystem manipulation study where the salinity of rock pools was increased at three different levels and decreased back to their original salinity levels after rainfall. The main aim was to determine how disturbance intensity, i.e., salinity of different intensities, affects bacterial communities in terms of composition, diversity and functioning, both at the local and regional scale. We specifically hypothesize (1) that compositional changes along the disturbance gradient are stronger than functional changes due to functional redundancy; and (2) that increasing salinity acts as a strong environmental filter and thereby reduces (a) alpha-diversity and (b) beta-diversity because deterministic community assembly processes are enforced.

## Materials and Methods

### Experimental Design

The experiment was performed in a rock pool area located on the island of Gräsö at the Baltic Sea coast of Sweden (latitude: 60.49824, longitude: 18.42900). The studied area contains approximately 250 pools of different sizes and different water characteristics and extends to approximately 325 m × 75 m (Supplementary Figure 1A and Table 1). On July 29, 2010, a survey of small to medium sized pools was conducted and a selection of freshwater pools was made. The selected pools (*n* = 27) differed slightly in size and volume, but also regarding other physical and chemical properties (Supplementary Table 1). All pools were sampled at three times points (Supplementary Figure 2) and the following parameters were measured: (i) BCC and diversity, (ii) bacterial abundance, (iii) environmental data including salinity/conductivity and chlorophyll *a* (Chl *a*), dissolved organic carbon (DOC), and total phosphorus (TP) concentrations and finally (iv) functional properties, such as carbon substrate utilization rate (CSUR) of the bacterial communities based on Biolog Ecoplates^TM^ and community respiration (oxygen consumption).

After selecting the pools, we performed the first sampling (T0) to determine initial, i.e., pre-disturbance, conditions. Following, we emptied each pool and filled the water into buckets in order to measure its volume. Depending on the salinity treatment we then returned different volumes of water to the pool basin of each pool (see below). Following, we added sea salt at four different salinity levels: control (no salt addition), 3 psu, 6 psu, and 12 psu. A previous experiment (Berga et al., 2012) has shown that these salinity levels reduce abundances and production rates of rock pool bacterioplankton from the same sampling area, which is why they were chosen to manipulate disturbance intensities in this study. The volume reductions compared to the initial volumes for the different salinity treatments were 5% for the 3 psu, 10% for the 6 psu, and 20% for the 12 psu treatment. These differences ensured that the salinity in the pools approached freshwater conditions (0 psu) once they were re-filled by rainwater inputs. Six pools were included for each treatment with exception of the controls that had nine pools, i.e., three pools for each volume reduction treatment to be able to control for the effect of volume reduction. Salt addition resulted in salinities between 2.6 and 3.4 for the 3 psu treatment, 5.8 and 6.2 for the 6 psu treatment, and between 10.4 and 14.5 for the 12 psu treatment. After the salt addition, all pools, including the controls, were covered to prevent dilution in case of a rain event. An emergency blanket was used in order to reduce warming of the pools as a result of the covering (Supplementary Figure 1B). After 65 h, we uncovered the pools and took samples to address the response of the bacterial communities to the salinity change (T65). After that, the pools remained open and we waited for rainfall to re-fill them. After a rain event of approximately 3 days and one additional day, i.e., 160 h after the initial sampling, initial conditions were regained and the experiment was ended. At this point, each pool was sampled again to assess the extent of recovery of the community (T160) (Supplementary Table 1).

### Sample Analyses

#### Sequencing and Sequencing Data Analyses: BCC and Community Diversity

Approximately 50–100 mL of each sample were filtered through 47 mm 0.2 μm Supor 200 filters (Pall Corporation, Port Washington, NY, United States). DNA extraction, 16S rRNA gene amplification and pre-sequencing processing of the PCR products were performed as in Berga et al. (2015). Amplicon sequencing was performed at the SNP&SEQ Technology Platform at Uppsala University using the 454 GS FLX pyrosequencing system (Roche/454) and Titanium chemistry (BIO Laboratories, Inc.). The sequences have been deposited in the NCBI Sequence Read Archive under accession number SRP065611.

A total of 916,239 sequences were obtained from the sequencing center. Denoising with Ampliconnoise (Quince et al., 2011) and chimera screening removed 14.3% of the initial sequences resulting in 11,285 operational taxonomic units (OTUs). OTUs were defined by clustering the sequences at a 97% similarity with the furthest-neighbor (complete linkage) algorithm using Qiime 1.6 (Caporaso et al., 2010). Following, the representative sequences for each OTU were blasted against the RDP library trained with the GreenGenes database in Qiime. After removing OTUs classified as eukarya, chloroplasts or archaea, 10,954 OTUs were left. For the statistical data analyses, sampling efforts (number of sequences obtained per sample) were normalized across the 81 samples by subsampling the data. A “subsampled OTU table” was created by performing 100 random subsamplings at 4,152 reads in R and then calculating the mean of each of the 100 subsampled tables. By subsampling at this level, we discarded four samples that had lower read numbers (Supplementary Table 1). The “subsampled OTU table” was then used for the calculation of community diversity and for all BCC analyses (see details below). Local diversity was estimated by calculating observed richness (S.Obs) and the Shannon index (*H*′) in each of the 100 randomized OTU tables and later calculating the average. Pileou’s evenness (*E*) index was calculated by *E* = *H*′/*H*_max_, where *H*_max_ = Ln(S.Obs).

#### Functional Parameters and Environmental Data

Community respiration was estimated using an optic syringe following details in Berga et al. (2015) over a 48 h period. CSUR was obtained as described in Berga et al. (2012) and environmental parameters TP, DOC, and Chl *a* were measured as in Langenheder and Ragnarsson (2007).

### Statistical Analyses

Statistical analyses were performed as follows: repeated measurement ANOVAs, one-way and two-way ANOVAs were performed in SPSS or R (R Core Team, 2016). Additional statistical analyses were performed using the “Vegan” (Oksanen et al., 2015), “car” (Fox and Weisberg, 2011), and “lme4” (Bates et al., 2012) packages in R.

#### Overall Patterns and Environmental Changes

To visualize overall changes in community composition in response to the experimental manipulation an NMDS based on Bray–Curtis (BC) dissimilarity was performed. To test whether volume reduction had an effect on community composition, we performed a PerMANOVA test on the controls at each sampling point using BC dissimilarity. To identify potential effects of the manipulation on environmental parameters, repeated measures ANOVA tests on log-transformed data were performed.

#### Time-Dependent Treatment Effects

Due to the nature of the experimental design, the different pools might, to some extent, not be appropriate replicates since they were all different from each other in the beginning. Thus, we analyzed time-dependent treatment effects by looking at changes of individual pools over time as an indicator of community turnover. To do this we calculated the difference between the measurements at T65 or T160 and T0 within a single pool for the following variables: S.Obs, *E*, and BC dissimilarity leading to the following new variables: ΔS.Obs_T65_, ΔS.Obs_T160_ Δ*E*_T65_, Δ*E*_T160_, ΔBC_T65_, and ΔBC_T160_. We performed the same calculation with the functional parameters resulting in ΔCSUR_T65_, ΔCSUR_T160_, ΔRespiration_T65_, and ΔRespiration_T160_. The Δ_T65_ values were used as proxy for resistance and Δ_T160_ as a proxy for recovery. Then log-transformed or sqrt-transformed absolute values were used in two-way ANOVA tests to identify patterns in the magnitude of change for the different parameters between salinity levels and over time (Δ_T65_ and Δ_T160_). For those parameters where significant effects of salinity or a significant interaction between salinity and time were observed, one-way ANOVA test and Bonferroni *post hoc* tests for each individual time point were performed to identify exactly which treatments differed from each other.

#### Changes in Main Bacterial Groups, Most Abundant OTUs, and Shifts between Abundant and Rare OTUs

Changes in the sequence abundance of major bacterial groups at the class level were studied by summing up the number of reads of all the OTUs belonging to the same class at each time point. We included only groups that had a relative abundance of at least 5% of the total community reads in at least one sample and investigated how these groups changed over time and according to the salinity treatment by means of a repeated measures ANOVA using sqrt- or log-transformed data. In the cases where the treatment or the interaction between treatment and time had significant effects, ANOVA tests were performed for each time point separately. We calculated the proportion of all OTUs that changed from being abundant to rare and vice-versa in response to the manipulation for each of the pools. The threshold for separating rare from abundant OTUs was set at 0.1% of the total reads, which is widely used to separate rare from abundant OTUs (Lynch and Neufeld, 2015). We tested if there were differences in the proportion of abundant and rare OTUs between the treatments with a one-way ANOVA on AsinSqrt-transformed data. Finally, we determined the most abundant OTUs in the entire dataset, defined as those with a number of reads ≥1% (including all the pools and time points). We then calculated the change in the number of reads for the most abundant OTUs between T65 and T0 to assess whether they were positively or negatively affected by the salinity change.

#### Assembly Mechanisms: Stochasticity vs. Determinism and Regional Processes

To determine whether the intensity of the salinity increase affected assembly mechanisms of the bacterial communities in the pools, we applied the null model approach based on the Raup–Crick metric (βRC) described in Chase (2007) and in Chase and Myers (2011) using the “Vegan package” in R. Raup–Crick results were obtained and analyzed as in Berga et al. (2015). Briefly, deterministic processes are indicated by βRC-values between -0.95 and -1 as well as values between 0.95 and 1. Any other value of βRC indicates that the communities are stochastically assembled. βRC metrics between pairs of communities were obtained after 100 iterations and was then recoded into binary data. Finally, the proportion of stochastically (0) and deterministically (1) assembled pairs of communities was calculated for the entire dataset. Differences in the proportion of deterministically assembled communities between salinity levels over time were then analyzed with a general linear model (glm in R) with binomial distribution followed by an ANOVA in R package “car.” In addition, differences in beta-diversity within each salinity treatment over time were tested by calculating BC dissimilarities between all possible pairwise combinations of pools belonging to the same treatment. We used a repeated measures ANOVA to test whether salinity and time had significant effects on average BC dissimilarities.

## Results

### Overall Patterns and Environmental Change

There were clear differences in BCC among pools at the beginning of the experiment (average BC dissimilarity at T0 = 0.86 ± 0.09) as well as changes in community composition over time within individual pools (Supplementary Figure 3). Volume reduction did not affect community composition of the control treatments at any of the time points as observed by PerMANOVA test (Supplementary Table 2). There were significant changes in DOC and TP over time (Supplementary Figure 4), which occurred in the form of minor increases after the manipulation (T65) and strong decreases at the end (T160) due to dilution caused by the rainfall (Supplementary Figure 4). On the other hand, Chl *a* concentrations did not change significantly over time. There were marginally significant differences in Chl *a* concentrations among treatments (Supplementary Figure 4).

### Time-Dependent Treatment Effects: Community and Functional Turnover

There were significant changes over time compared to initial values in many of the studied metrics, whereas effects of salinity were only significant in cases of BC dissimilarities (**Table 1**). Moreover, significant interactive effects between salinity level and time, were found for BC similarities and CSURs, indicating that the degree of change of the parameters at T65 and T160 differed between salinity treatments.

**Table 1:** Results from two-way and one-way ANOVAs testing the effect of salinity on differences in species richness (ΔS.Obs), evenness (Δ*E*), Bray–Curtis dissimilarity (ΔBC), carbon substrate utilization rate (ΔCSUR), and respiration rate (ΔResp) between T0 and T65 and T0 and T160, respectively for each pool.

Two-way ANOVA						
	**Time**		**Treatment**		**Interaction**	
	***F*-value**	***p*-value**	***F*-value**	***p*-value**	***F*-value**	***p*-value**

ΔS.Obs	**12.43**	**0.01**	1.66	0.19	0.43	0.73
ΔE	3.3	0.08	0.31	0.82	0.9	0.45
ΔBC	**5.43**	**0.03**	**4.19**	**0.01**	**7.03**	**0.01**
ΔCSUR	**19.71**	**0**	0.98	0.41	**5.47**	**0.00**
ΔResp	0.12	0.73	1.62	0.20	0.34	0.79

**One-way ANOVA (treatment)**						

	***F*-value**	***p*-value**		***Post hoc* grouping**	

ΔBC65	**9.34**	**0.001**		**6 ≠ 0, 12 ≠ 0, 12 ≠ 3**	
ΔBC160	0.80	0.51			
ΔCSUR65	**4.22**	**0.02**		**12 ≠ 0, 12 ≠ 3, 12 ≠ 6**	
ΔCSUR160	1.63	0.22			

*Bold values highlight *p*-values <0.05. T, time; S, salinity. One-way ANOVA and *post hoc* tests were performed if salinity or the interaction between salinity and time was significant in the two-way ANOVAs. Salinity treatment levels in *post hoc* tests are indicated as: 0, control; 3, 3 psu; 6, 6 psu; 12, 12 psu.*

**FIGURE 1:**
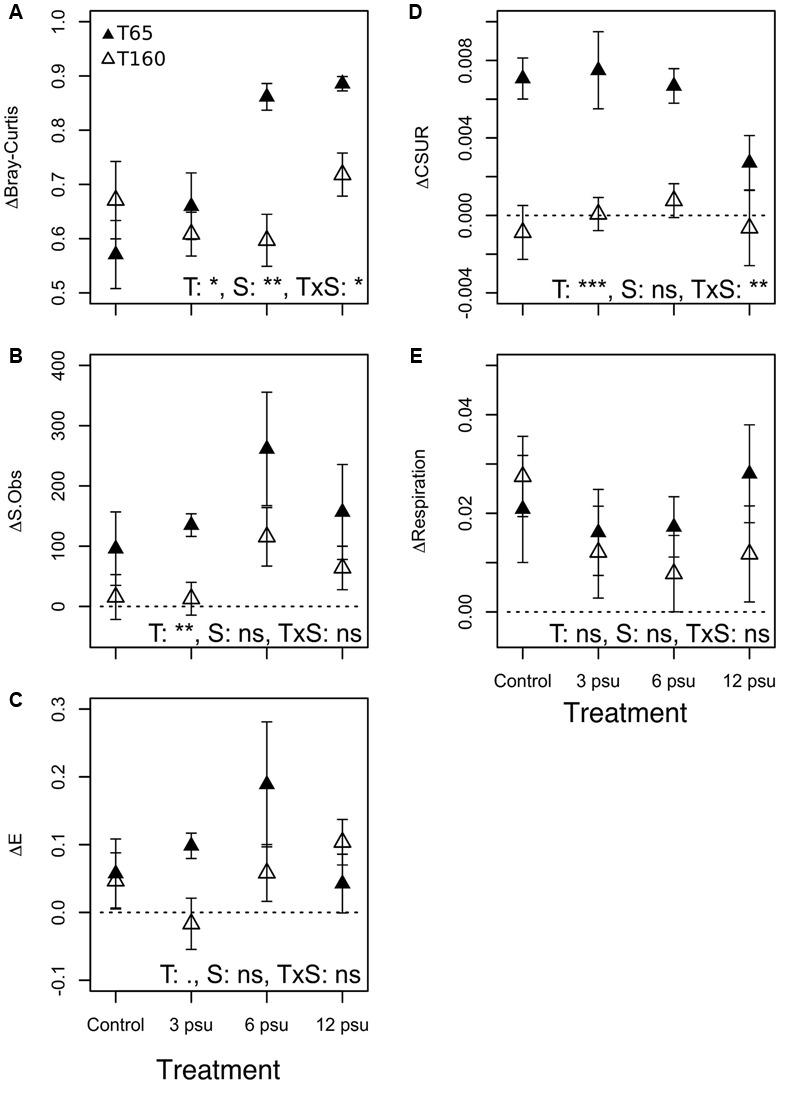
**Changes in (A)** community composition (ΔBray–Curtis), **(B)** species richness (ΔS.Obs), **(C)** evenness (Δ*E*), **(D)** carbon substrate utilization rates (ΔCSUR), and **(E)** respiration rates (ΔRespiration) between T0 and T65 (filled symbols) as measurement of resistance and between T0 and T160 (empty symbols) as a measurement of resilience at the different salinity levels. Dashed lines indicate the “0 value” (no change). Error bars indicate standard errors. ANOVA results are indicated as follow: T, time; S, salinity; T × S indicates the interaction. ^∗∗∗^*p* < 0.001, ^∗∗^*p* < 0.01, ^∗^*p* < 0.05, ^•^*p* < 0.1, and ns, not significant.

BC dissimilarities showed that BCC in the pools differed the most compared to their initial community at T65 but became more similar to the initial composition at T160, showing a certain degree of recovery (**Figure 1A**, Supplementary Figure 3, and **Table 1**). Additionally, there were significant differences in the magnitude of change from the initial community composition between the different treatments at T65. Specifically, pools that received 6 and 12 psu of salt differed more from their original composition than the control pools and low salinity pools did. However, at T160 there were no significant differences between the treatments (**Figure 1A** and **Table 1**).

In terms of diversity, both S.Obs and evenness increased in most of the pools at T65 compared to the initial values. This increase was observed in almost all treatments including the controls, and was slightly, but not significantly stronger in the pools that received salt. Richness and evenness decreased and approached original levels at T160 in most cases (**Figures 1B,C** and **Table 1**). The exception to this was observed in the case of evenness in the 12 psu treatment, which showed the strongest increase compared to the original community at T160. CSURs were much higher at T65 than initially, but this increase was lower in the 12 psu salinity compared to the other treatments. At T160, CSURs had recovered to almost identical values than at T0 and there were no differences between the treatments (**Figure 1D** and **Table 1**). Finally, respiration rates were unaffected by the manipulation and did not change significantly compared to initial rates nor between salinity treatments (**Figure 1E, Table 1**, and Supplementary Figure 6).

### Changes in Main Bacterial Groups

**FIGURE 2:**
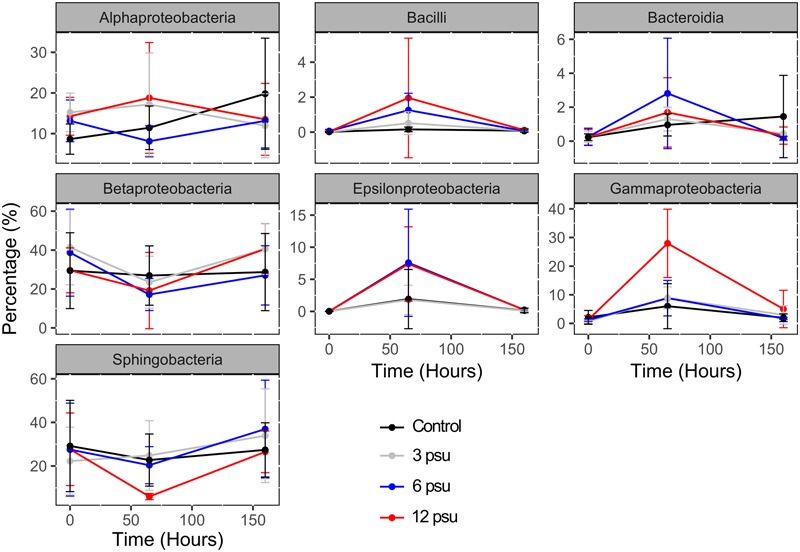
**Changes in the relative abundance of sequences associated to the main bacterial phyla, families and genera over time and between salinity levels.** Error bars indicate standard errors.

From the 15 bacterial groups included in the analyses, seven showed significant changes in their sequence abundances over time in response to experimental manipulation (**Figure 2** and Supplementary Table 3). Moreover, for Bacilli, Gammaproteobacteria, and Betaproteobacteria the temporal changes in sequence abundances differed depending on salinity. While Bacilli, Bacteroidia, Gammaproteobacteria, and Epsilonproteobacteria increased at T65 and then decreased at T160 in all treatments particularly in the high salinities treatments (**Figure 2**), the opposite pattern, i.e., significantly lower relative abundances at T65 compared to T0 and T160 was found for the Betaproteobacteria and Sphingobacteria (**Figure 2** and Supplementary Table 3). When analyzing the proportion of OTUs that switched from being abundant (≥0.1% of the total reads) to rare (<0.1%), and vice versa, we found that 3–6% of the OTUs in each pool switched from being common to rare. On the contrary, OTUs that switched from being rare to abundant ranged between 7 and 10% (Supplementary Figure 5). There were no significant differences in the proportion of these two classes of OTUs between treatments, however, we did observe a significantly higher number of OTUs switching from rare to abundant than vice versa in treatments with low and intermediate salinities (3 and 6 psu; Supplementary Figure 5). The most abundant OTUs (>1%) in the entire dataset included 15 OTUs that all belonged to Bacteroidetes and Proteobacteria and were negatively affected by the mixing caused by the manipulation. These most abundant OTU included both sensitive types that decreased in response to one or several salinity levels, as well as tolerant OTUs whose abundance remained unchanged (Supplementary Table 4).

### Regional Changes and Changes in Assembly Mechanisms

**FIGURE 3:**
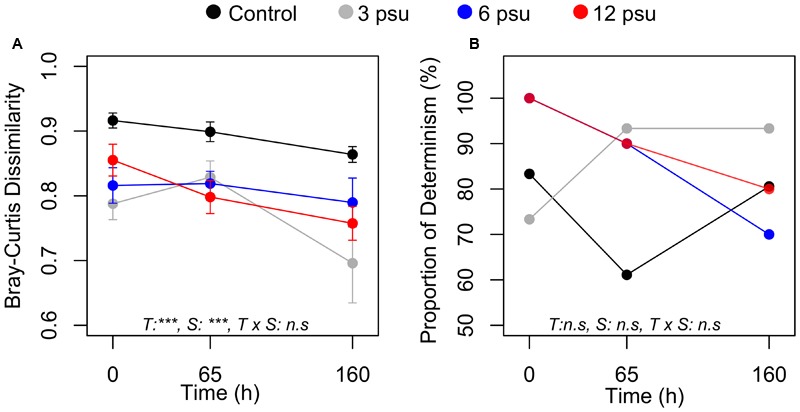
**Changes in beta-diversity based on the average Bray–Curtis dissimilarity (A)** and proportion of deterministically assembled pairs of communities (proportion of determinism) **(B)** over time and between salinity levels. Error bars indicate standard error. ANOVA results are indicated as follow: T, time; S, salinity; T × S indicates the interaction. ^∗∗∗^*p* < 0.001, ^∗∗^*p* < 0.01, ^∗^*p* < 0.05, and ns, not significant.

Average pairwise BC similarity, i.e., beta-diversity, changed significantly over time and differed significantly between the treatments at all time points. Beta-diversity was lowest for most of the salinity treatments at T160 and generally highest in the control treatment (**Figure 3A**). Bacterial communities in rock pools communities were predominantly deterministically assembled (**Figure 3B**) and there were neither significant differences over time nor between treatments in the proportion of deterministically assembled communities (**Figure 3B**).

## Discussion

This study employed whole ecosystem manipulations to investigate the mechanisms by which freshwater bacterial communities responded to environmental change, specifically increasing salinities, at local as well as regional scales, and aimed to identify whether functional and compositional recovery occurs. The major finding was that community composition is the most sensitive parameter to salinity changes, whereas community functions (respiration and CSUR) did not change. These results support our first hypothesis because compositional resistance decreased with increasing salinity levels, whereas functioning was stable due to functional redundancy. However, community composition showed a high level of recovery, i.e., became similar to pre-disturbance conditions once salinity changes were reversed. On the contrary, the second hypothesis had to be rejected because alpha-diversity (richness and evenness) and beta-diversity were resistant to any of the applied levels of salinity change.

Our study is in line with results of previous lab-based studies that have shown that the intensity of a disturbance or environmental change determines its effect on BCC (Ager et al., 2010; Berga et al., 2012). Hence, BCC changed at an increasing magnitude with increasing salinities. Moreover, we found changes in the relative abundance of major bacterial classes reflecting a shift from those with preferences for freshwater vs. marine conditions with increasing salinities in the pools (Zwart and Crump, 2002; Newton et al., 2011). For example, Betaproteobacteria that are typically more abundant in freshwater compared to marine environments decreased after salt additions (Nold and Zwart, 1998; Newton et al., 2011), whereas Gammaproteobacteria that are often found in lower abundances in freshwater compared to marine environments (Barberán and Casamayor, 2010; Herlemann et al., 2011), increased in response to salt additions. On the contrary, increasing salinities did not change broad bacterial community functions such as respiration rates or the rate of carbon utilization (CSURs), which were also found to be resistant or resilient to salinity or nutrient disturbances in previous studies (e.g., Bowen et al., 2011; Baho et al., 2012; Berga et al., 2012). Functional redundancy may, however, depend on the type and specificity of a function (Langenheder et al., 2006; Peter et al., 2011), so that we cannot preclude that different results, including concomitant changes in community composition and functioning along disturbance gradients, are found if different functions are studied.

An interesting result of this study is the observed resistance of bacterial communities to small increases of salinity such as 3 psu. Resistance of BCC to disturbances has been observed in other studies (e.g., Bowen et al., 2011), however, here it was surprising since previous results from laboratory experiments have shown that salinity increases of a similar magnitude can induce changes in BCC (Langenheder et al., 2003; Berga et al., 2012; Székely et al., 2013). Our findings here are, however, in congruence with a study that has been implemented along the salinity gradient in the Baltic Sea, where communities that were found in localities of salinities up to 3.2 psu were compositionally very similar to those found at 0 psu (Herlemann et al., 2011). In general, the resistance to small increases in salinity could be related to adaptation to fluctuating environmental conditions that include recurrent changes in salinity in rock pools. Support for this idea comes also from the observed high recovery capacity of the communities that encountered a salinity change of 6 and 12 psu. Shade et al. (2011) also reported high recovery rates after a disturbance, more specifically to water mixing, in aquatic bacterial communities and Girvan and Campbell (2005) observed recovery of soil bacteria after copper additions. It has been suggested that the disturbance history of a community can also influence its sensitivity and resilience to additional disturbances (Hawkes and Keitt, 2015). Additionally, it has also been shown that bacterial communities are less affected by strong disturbances when they have experienced smaller disturbances before (Bressan et al., 2008) or even that some bacterial strains can anticipate environmental changes because of pre-exposure to the disturbance (Mitchell et al., 2009). Hence, “stress priming” may be commonly found in bacterial communities (Rillig et al., 2015; Andrade-Linares et al., 2016), in particular also in response to osmotic stress (Andrade-Linares et al., 2016). Rock pools are small and often temporary ecosystems that show strong fluctuations in environmental conditions over time. Thus, it seems possible that their disturbance history and historical environmental filtering could explain why bacterial communities were relatively little affected by salinity. In our studies, the communities did not change unless they experienced strong increases in salinity (6 and 12 psu) which they have probably rarely experienced before.

The fact that we did not observe any negative effect of increasing salinities on species richness could be related to the magnitude of the change, which might not have been strong enough to suppress or filter out many bacterial species. Herlemann et al. (2011) showed that, opposite to what is expected for macroorganisms, where richness is lowest at intermediate salinities, i.e., brackish conditions (Telesh and Khlebovich, 2010), increases in salinities did not affect bacterial species richness. This could be a result of a fast adaptation of bacteria to brackish conditions, for example, by generalist taxa (such as OTUs 1659 and 10659 which were unaffected by salinity increases and abundant in all treatments; Supplementary Table 4). Alternatively, fast replacement of sensitive bacteria with opportunists could also explain why we did not observe decreases in species richness with increasing salinity in our study. Support for this comes from the observation that a considerable number of OTUs switched from being rare to abundant after the disturbance, and, vice versa, there was a similar number that “switched” in the other direction. Likewise, beta-diversity did not decrease along the disturbance intensity gradient, which suggests that the applied salinity changes were not strong enough to decrease variability among local communities due to the filtering imposed by the disturbance as has been observed in animal metacommunities (Chase, 2007; Lepori and Malmqvist, 2009). This seems likely because the importance of deterministic vs. stochastic assembly processes did not change along the disturbance gradient. The overall dominance of deterministically assembled communities that we observed suggests that environmental selection is the major structuring factor in the pools as also seen in Langenheder and Ragnarsson (2007), Langenheder et al. (2012), Székely and Langenheder (2014), whereas stochastic (neutral) assembly processes are relatively less important than in other types of aquatic ecosystems (Östman, 2011).

The experimental manipulation included the coverage of the pools during the disturbance period and an initial mixing of water that led to changes in other environmental parameters such as DOC and TP concentrations. This occurred equally in all treatments, including the controls, and may explain some of the temporal patterns that we observed in all pools irrespective of the salinity level. Firstly, the pattern observed for species richness, with higher values at T65 and close to initial values at T160 could be explained by the manipulation of the pools. For example, the mixing of the pool water, which resulted in the re-suspension of precipitated material and bacteria found in the sediments, could have “seeded” the water column and thereby induced an increase of richness in all the pools. On the other hand, another possible explanation for this pattern could be that light exclusion, due to the covering of the pools, affected primary producers in the pools. This seems unlikely because Chl *a* concentration did not decrease during the period when the pools were covered, however, stress responses of phototrophic organisms, such as changes in their heterotrophic activity (mixotroph organisms) or formation of resting stages might have directly affected the bacterial communities, and in particular evenness, by alterations in intra-specific competition with autotrophs (McKnight et al., 2000; Eiler, 2006; Hornák et al., 2006). The increase in CSURs after the manipulation and subsequent return to initial levels by the end of the experiment, could also have been caused by increases in nutrient concentrations and substrate diversity due to the water mixing that may have interfered with or facilitated the use and/or rates of use of the carbon substrates in the Biolog Ecoplates. An alternative explanation is that the increase in CSUR is related to functional complementarity due to increases in species richness (Venail et al., 2008), which was also found in response to the manipulation, supporting previous findings of positive relationships between bacterial richness and ecosystem functions (e.g., Bell et al., 2005; Langenheder et al., 2010; Peter et al., 2011).

To summarize, our results show that freshwater bacterial communities were (a) functionally resistant to salinity changes and (b) compositionally resistant to salinity changes up to 3 psu and resilient to salinities ≥6 psu. OTU richness, evenness, as well as beta-diversity or the underlying community assembly mechanisms seemed to be unaffected by increases of salinity. More studies are needed to evaluate whether our findings generally apply to different types of natural systems or are specific for environments with strongly fluctuating environmental conditions and strong legacy effects, such as different types of small and temporary aquatic habitats. Future studies should therefore aim to understand how the perturbation history of communities influences their functional and compositional resilience at local and regional scales. To conclude, the important finding from our whole ecosystem manipulation experiment is that the stability of bacterial communities was generally high compared to many previous studies carried at the laboratory scale (e.g., Shade et al., 2012).

## Author Contributions

MB designed and performed the experiment, samples processing, data processing, and writing. YZ and AS performed the experiment, sample processing, and writing. SL designed and performed the experiment, samples processing, writing, and financial support.

## Conflict of Interest Statement

The authors declare that the research was conducted in the absence of any commercial or financial relationships that could be construed as a potential conflict of interest.
