# Supplementary material for: Functional and Compositional Stability of Bacterial Metacommunities in Response to Salinity Changes
Source: Front Microbiol. 2017 Jun 8;8:948. doi: 10.3389/fmicb.2017.00948 (PMC5463035; doi:10.3389/fmicb.2017.00948)
Supplement: Supplementary file 1 [file Data_Sheet_1.pdf]

Supplementary Material

Mercè Berga[*], Yinghua Zha, Anna J. Székely and Silke Langenheder

*** Correspondence:** merce.berga@io-warnemuende.de

1 Supplementary Figures and Tables

1.1 Supplementary Figures

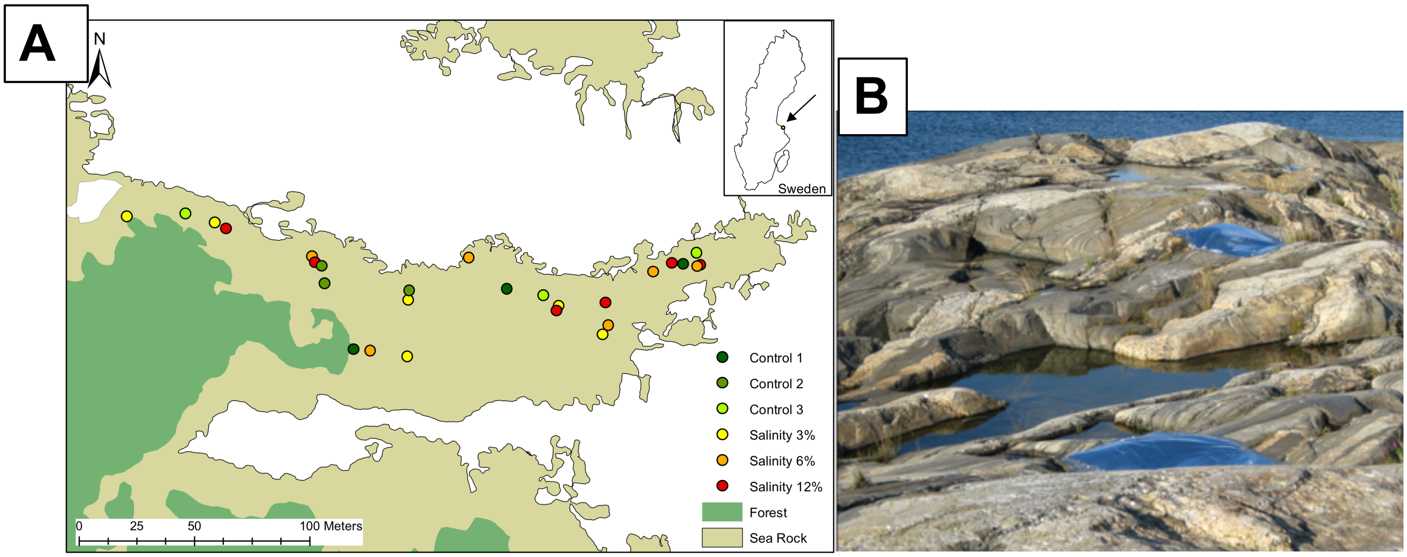

Supplementary Figure 1. A- Map of the area and distribution of the pools and their respective treatments. © Lantmäteriet Gävle (2010): Permission I 2010/0058. B- Picture from the rock pool area during the experiment where covered pools can be seen.

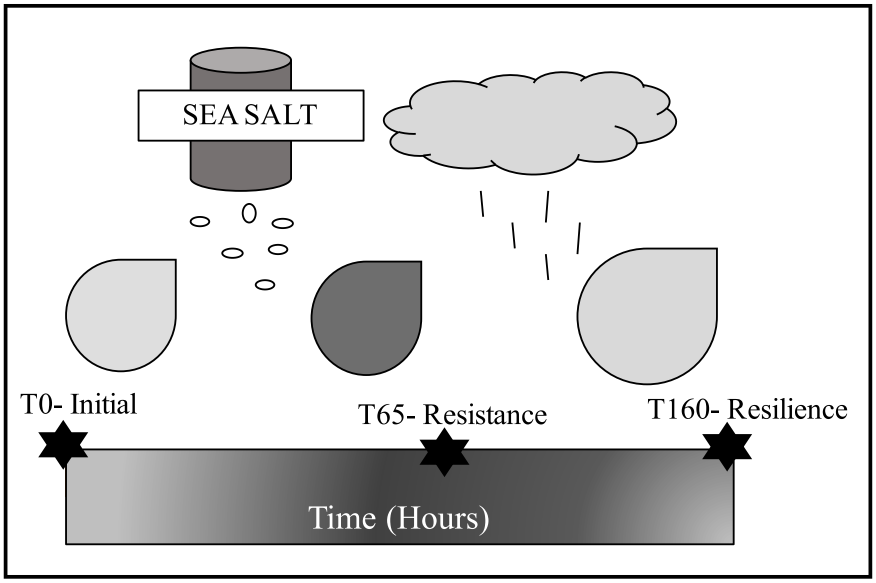

Supplementary Figure 2. Schematic representation of the experimental set-up.

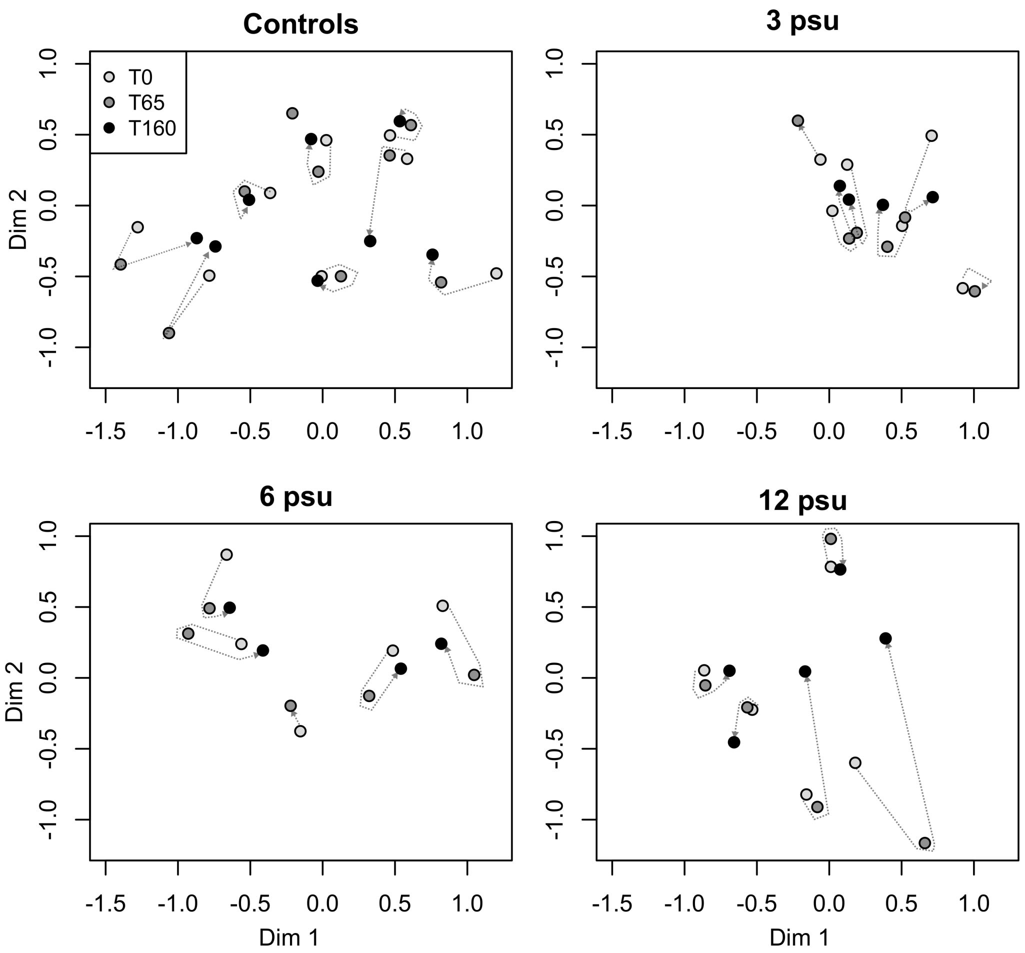

Supplementary Figure 3. NMDS graphs showing differences in bacterial community composition at the different time points and salinity levels. K-stress = 0.191. Discontinuous arrows indicate the shift over time.

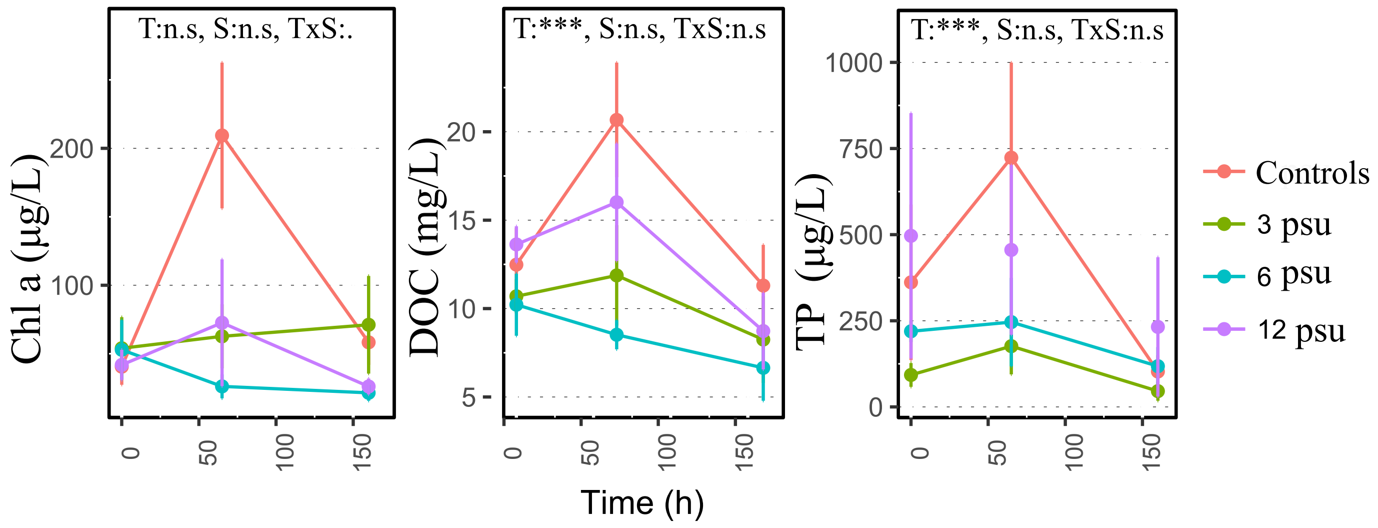

Supplementary Figure 4. Chlorophyll a, DOC and total phosphorus (TP) concentration at each time point in the different salinity treatments. Error bars indicate standard errors. ANOVA results are indicated as follows: T - time, S - salinity, T x S indicates the interaction. *** : $p < 0.001$, ** : $p < 0.01$, * : $p < 0.05$, . : $p < 0.1$ and n.s : not significant.

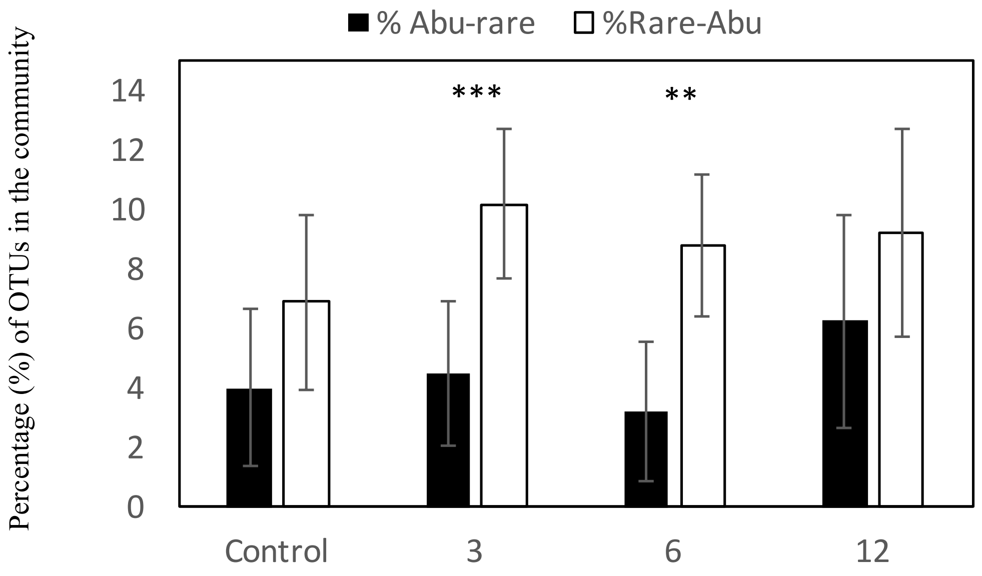

Supplementary Figure 5. Proportion of OTUs changing from abundant to rare and vice-versa in the pools of the different salinity treatments (3- 3 psu, 6- 6 psu and 12- 12 psu). Error bars indicate standard deviation and stars indicate the level of significance between the two categories according to a t-test: *** - $p < 0.001$ and ** - $p < 0.01$.

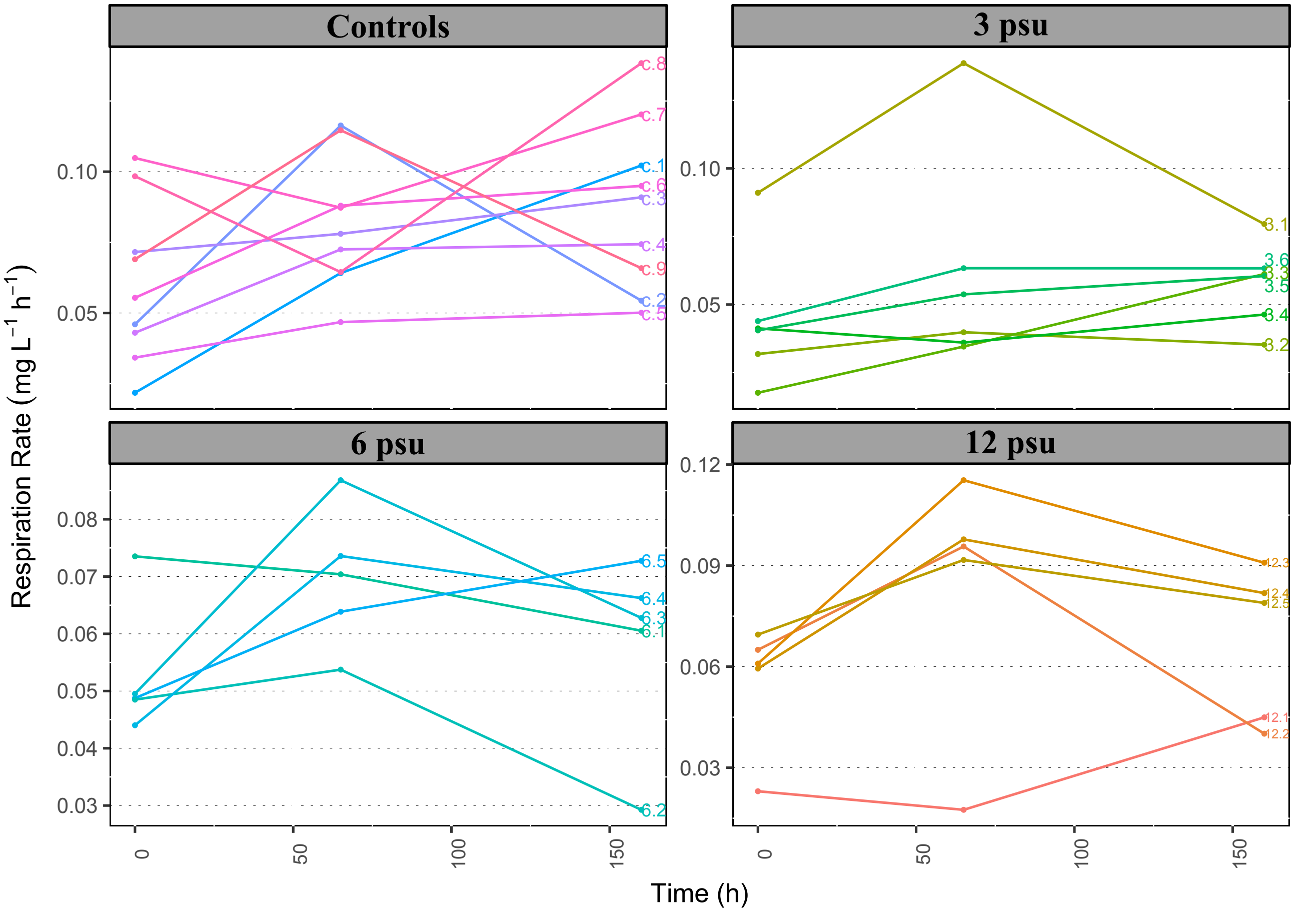

Supplementary Figure 6. Plots showing the variability of community respiration rates for each of the pools within each treatment over time. Each line represents a pool and the names of the pools are shown at the end of the line.

1.2 Supplementary Tables

Supplementary Table 1. Physical and environmental characteristics of the pools at the beginning of the experiment. Abbreviations: Conduct. - conductivity, Est. vol. - Estimated volume, Chl a – Chlorophyll a concentration, DOC – Dissolved organic carbon concentration, TP – Total phosphorus concentration.

Pool	Original Salinity	Original Conduct.	Length (cm)	Width (cm)	Depth (cm)	Est. vol. (cm^3)	Chl a (µg/L)	DOC (mg/L)	TP (µg/L)
c.1	ofl	54.7	220	51	15	56100	16.11	21.06	73.87
c.2	ofl	57.3	282	65	11	67210	27.25	10.31	438.65
c.3	ofl	161	186	57	25	88350	125.77	9.67	67.67
c.4	ofl	27.8	110	62	9.5	21597	25.94	9.17	54
c.5**	ofl	20.6	225	51	10	38250	10.66	7.57	14.99
c.6	ofl	24.8	239	28	8	17845	8.53	9.03	41.3
c.7	ofl	51.2	130	100	16	69333	83.22	24.04	55.57
c.8	0.1	337	188	63	15	59220	48.72	15.11	2118
c.9	ofl	43.1	126	49	8	16464	18.48	6.39	390.85
3.1	ofl	55.3	135	65	14	40950	146.9	13.24	205.42
3.2	ofl	49.7	165	45	22	54450	2.25	11.22	58.64
3.3**	ofl	30.3	263	102	18.5	165427	33.05	12.96	22.34
3.4	ofl	35.6	160	49	22	57493	6.99	9.48	14.57
3.5	NA	NA	126	36	9	13608	91.57	6.91	64
3.6**	ofl	45	212	206	9	131016	43.12	10.36	191.75
6.1	ofl	27.7	230	50	13	49833	43.71	6.91	61.71
6.2	ofl	22.9	168	118	11	72688	120.36	9.03	54.55
6.3	ofl	168	171	89	12	60876	3.2	17.04	452.2
6.4**	ofl	39.4	175	100	11.5	67083	14.66	9.07	35.76
6.5	ofl	106.2	166	94	8.5	44211	82.33	9.04	493.14
6.6*	0.1	334	104	40	14	19413	35.07	9.94	74.29
12.1	ofl	66	150	77	23	88550	16.59	10.52	37.51
12.2	ofl	55.5	170	80	12	54400	17.65	16.66	32.51
12.3	140	NA	185	64	9	35520	74.4	12.51	228.24
12.4	ofl	118.9	295	89	13	113772	52.72	13.87	277.36
12.5	0.1	385	130	83	17.5	62942	47.74	14.59	1907.4
12.6*	ofl	905	129	57	22	53922	23.34	6.27	44.67

*- Excluded from analyses because of marine infiltration during the experiment, **- One or more time points might have been excluded from some analyses because of low sequences number and **NA**- Missing value.

Table S2. Results from a PerMANOVA testing the effect of volume reduction on community composition of the control treatments at the different time points. Bold values highlight p-values < 0.05.

**PerMANOVA**		
	pseudo-F	p-value
T0	1.118	0.238
T65	0.999	0.422
T160	1.141	0.205

Table S3. Results from repeated measurements ANOVAs testing effects of salinity on the abundance of the main bacterial phyla over time and depending on the salinity levels. Bold values highlight p-values < 0.05. Transf. - Data transformation applied for ANOVA test

	**Time**	**Salinity**	**Time x Salinity**	**Transf.**
Acidobateria	F= 0.84, p= 0.44	F= 0.88, p= 0.47	F= 2.06, p= 0.09	Sqrt
Actinobacteria	F= 0.30, p= 0.75	F= 1.03, p= 0.40	F= 0.38, p= 0.89	Log
Alphaproteobacteria	**F= 5.06, p= 0.01**	F= 2.00, p= 0.15	F= 0.87, p= 0.53	Sqrt
Bacilli	**F= 25.73,**	**F= 2.98, p= 0.06**	F= 1.70, p= 0.16	Log
Bacteroidetes	**F=17.55, p< 0.001**	F= 0.51, p= 0.68	F= 0.36, p= 0.89	Log
Betaproteobacteria	**F= 6.38, p= 0.005**	F=0.57, p= 0.64	**F= 1.47, p= 0.22**	Sqrt
Deltaproteobacteria	F= 3.04, p= 0.06	F= 1.27, p= 0.31	F= 0.76, p= 0.61	Log
Epsilonproteobacteria	**F=61.27, p< 0.001**	F= 2.26, p= 0.11	F= 1.52, p= 0.21	Log
Flavobacteria	F= 0.63, p= 0.54	F= 0.74, p= 0.54	F= 1.20, p= 0.33	Sqrt
Gammaproteobacteria	**F=29.45, p< 0.001**	**F= 5.58, p= 0.006**	**F= 4.17, p=0.003**	Sqrt
Gemmatimonadetes	F= 1.45, p= 0.25	F= 0.82, p= 0.50	F= 0.78, p= 0.59	Sqrt
Oscillatoriophycideae	F= 0.96, p= 0.40	F= 1.34, p= 0.29	F= 1.34, p= 0.27	Sqrt
Sphingobacteria	**F= 7.05, p= 0.003**	F= 0.68, p= 0.57	F= 1.57, p= 0.19	Sqrt
Synechococcophycideae	F= 0.60, p= 0.56	F= 0.12, p= 0.94	F= 1.06, p= 0.41	Sqrt
Verrucomicrobiae	F= 0.22, p= 0.80	F= 0.19, p= 0.90	F= 0.38, p= 0.89	Log

Supplementary Table 4: List of the most abundant OTUs (> 1% of total of reads) and their taxonomic classification in the dataset. The average change in the number of reads between T0 and T65 of pools within each salinity treatment is also shown (12 – 12 psu, 6- 6 psu, 3- 3 psu and Cont- Control). Response strategies refer to sensitive OTUs that decreased in abundance when exposed to an increasing salinity compared to their initial abundance or, alternatively, OTUs that were unaffected by the salinity change. To define a sensitive response to salinity, the average change in reads of a specific OTU were more strongly negatively affected compared to the control. OTUs were classified as either "sensitive to all salinities", "sensitive to high salinity", "unaffected" and "undetermined" if no clear pattern was found.

OTU		12	6	3	Controls	Response Strategy
6928	Betaproteobacteria_ Polynucleobacter	-401,92	-721,43	-80,83	-86,04	Sensitive to high salinity
7929	Unclassified_bacteria	-25,16	-167,06	-150,93	-18,40	Undetermined
1011	Bacteroidia_ Marinilabilaceae	-129,76	123,08	139,29	-117,25	Undetermined
2298	Betaproteobacteria_ Comamonadaceae	-221,54	-55,91	-443,24	-43,97	Undetermined
2426	Betaproteobacteria_ Undefined Burhkholderiales	-98,68	-84,25	-123,52	-84,2	Undetermined
6311	Flavobacteria_ Flavobacterium	-155,7	-12,44	-137,71	1,38	Sensitive to salinity
587	Bacteroidetes_ Spirosoma	-30,24	-246,63	-108,06	-227,45	Undetermined
8591	Betaproteobacteria_ Comamonadaceae	-20,44	-36,46	-176,51	-52,10	Undetermined
4005	Flavobacteria_ Flavobacterium	-27,03	-77,69	-3,27	-163,74	Undetermined
6333	Alphaproteobacteria_ Rhodobacteraceae	-102,88	29,60	18,26	9,46	Sensitive to high salinity
1659	Gammaproteobacteria_ Aeromonas	609,62	107,93	61,74	18,02	Unaffected
4703	Bacteroidetes_ Chitinophagaceae	-337,85	-126,48	-109,37	-9,55	Sensitive to salinity
6804	Alphaproteobacteria_ Novosphingobium	-260,82	-24,06	-4,76	-85,50	Undetermined
3698	Bacteroidetes_ Cytophagaceae	-0,31	12,14	-119,03	-0,60	Undetermined
10659	Flavobacteria_ Chrysobacterium	308,65	137,50	116,62	4,88	Unaffected
